# Has Living on Islands Been So Simple? Insights from the Insular Endemic Frog *Discoglossus montalentii*

**DOI:** 10.1371/journal.pone.0055735

**Published:** 2013-02-05

**Authors:** Roberta Bisconti, Daniele Canestrelli, Giuseppe Nascetti

**Affiliations:** Dipartimento di Scienze Ecologiche e Biologiche, Università della Tuscia, Viterbo, Italy; Institut de Biologia Evolutiva - Universitat Pompeu Fabra, Spain

## Abstract

Island populations have been extensively used as model systems in ecology, biogeography, conservation and evolutionary biology, owing to the several simplifying assumptions that they allow. Nevertheless, recent findings from intra-island phylogeographic studies are casting doubts on the generality of some of these underlying assumptions. We investigated the phylogeography, historical demography, and population genetic structure of the Corsican endemic frog, *Discoglossus montalentii*. In contrast with expectations based on its insular, restricted and continuous distribution, we found evidence of 3 phylogroups, whose rather ancient divergence (Early-Middle Pleistocene) was likely primed by climatic changes that occurred during the ‘middle Pleistocene revolution’. Furthermore, their differentiation explained most (68%) of the overall genetic diversity that was observed. These results and the growing evidence from intra-island phylogeographies, suggest that island populations frequently may not conform to some long-standing assumptions, including long-term stability, range-wide panmixia and the correlation of effective population size to the island size. As a consequence, both for theoretical and for applied purposes, the extensive use of these assumptions in the study of island populations warrants a careful re-examination.

## Introduction

Islands are among the most important biodiversity hotspots on earth [1] and they are particularly interesting from many standpoints [2]. Endemism richness on islands surpasses that of the continents, with estimates more than 8 times higher for plant and vertebrate species [1]. Furthermore, islands have a longstanding history as ‘natural laboratories’ for the study of evolutionary and ecological processes [2]–[7]. Indeed, island populations have often been considered as simplified systems, relative to continental populations, with levels of genetic diversity and effective population sizes correlated to island size, degree of isolation, and tempo and mode of island occupancy [2], [6], [8]–[11]. This assumption is commonly applied in empirical and theoretical studies, with far reaching implications for ecological, evolutionary and conservation perspectives (see also [12] for a lengthier discussion on this issue). Nevertheless, a growing amount of literature is challenging this perception. In particular, intra-island phylogeographic studies are revealing complex population structures that are far from conforming to an expectation of island-wide panmixia (see e.g. [12]–[17]). On the whole, these cases offer an invitation to extend our current knowledge on the structure of diversity of island populations, and the relevance of micro-evolutionary processes acting within single islands in shaping current patterns of diversity. In doing so, we can further aim to assess the plausibility of the simplifying assumptions commonly made about island populations.

The island of Corsica is one of the more than 5000 islands in the Mediterranean basin [18]. It is the northernmost, the most mountainous, and the wettest island of the basin. It has a particularly high diversity of ecosystems and climates, from a Mediterranean climate at low altitudes to an alpine climate at higher altitudes (see [19] for an extensive description of the Corsican climates and environments). Moreover, it is a hotspot of Mediterranean biodiversity, with many species being endemic [19]–[20]. Recently, several phylogeographic studies have been carried out on species from this island, including amphibians, reptiles, invertebrates, and plants [12], [21]–[28]. These studies have identified a diversity of genetic patterns and evolutionary histories, including evidence of both shallow and deep population fragmentation (e.g. [12], [22], [26], [27]).

The Corsican painted frog, *Discoglossus montalentii*, is a species endemic to the mountain regions of the island of Corsica, specifically in the central portion of the island. Once attributed to the Tyrrhenian Painted frog *D. sardus*, populations of *D. montalentii* were then attributed to a separate species based on studies of their allozyme variation [29]. Subsequent phylogenetic studies also identified this species as a lineage of ancient derivation within the genus *Discoglossus* ([30] and references therein]. *D. montalentii* lives in fresh and running waters, especially in streams traversing woods and forests. Its altitudinal range varies from 300 to 1900 m above sea level, and it is absent from coastal lowlands. Although little is known about the conservation status and population dynamics of this species [31], it is believed to be declining due to fish introductions, and it is listed as ‘Near Threatened’ in the IUCN Red List of Threatened Species, based on the criteria for this designation, namely that “…its range is less than 5000 km^2^ and the species is not considered to be highly fragmented” [31]. Moreover, it is listed on Appendix II of the Bern Convention and on Annexes II and IV of the EU Habitats Directive.

In this study, we investigated the mtDNA genetic variation of *D. montalentii*. Our aims were to gain insight into the phylogeographic structure and evolutionary history of *D. montalentii* and to contribute to shedding more light on the micro-evolutionary processes involved in generating current patterns of genetic diversity within island species. We therefore carried out phylogeographic historical demographic and population genetic structure analyses based on 2 mitochondrial DNA gene fragments. Finally, since no data about the population structure and diversity of this species are yet available, we also evaluated the significance of our results for the species' conservation.

## Materials and Methods

### Ethics statement

*Discoglossus montalentii* individuals were captured under a permit from the Direction Régionale de l'Environnement, de l'Aménagement et du Logement (DREAL) de Corse. Frogs were captured with nets at their breeding sites. We collected tissue samples from toe-tips after anaesthetizing frogs by submerging them in a 0.1% solution of MS222 (3-aminobenzoic acid ethyl ester). No individuals were brought to the laboratory or sacrificed. After the completion of the sampling procedure, all the individuals were immediately released at the collection site. Tissue samples were stored in 96% ethanol until further analyses.

### Laboratory procedures

**Figure 1:**
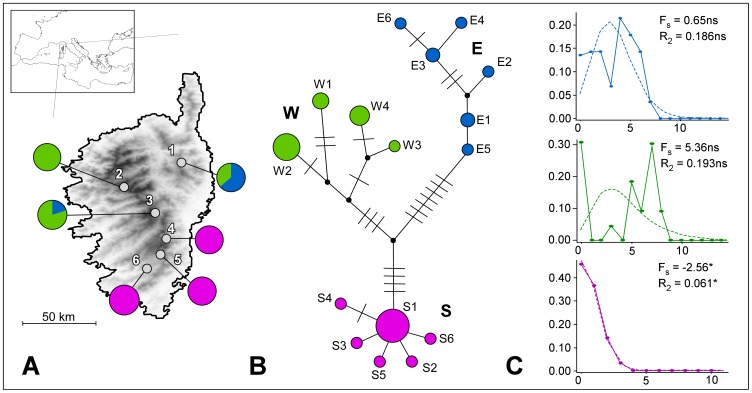
Geographic distribution of the 6 sampling sites of *Discoglossus montalentii*, phylogenetic relationships of the 16 haplotypes found and historical demography of the main haplogroups. (A) Geographic location of the 6 sampled populations of *D. montalentii*, and frequency distribution of the main haplogroups, shown as pie diagrams. Populations are numbered as in Table 1. (B) Median-joining network depicting the phylogenetic relationships among all mtDNA haplotypes found. Circle sizes are proportional to haplotype frequency in the dataset; missing intermediate haplotypes are shown as lines while median vectors are shown as black points. (C) Mismatch distributions and values of the demographic test statistics for the 3 main clades identified by the network analysis. *F*_S_: Fu's *F*_S_ statistic [44]. *R*_2_: Ramos-Onsins & Rozas' *R*_2_ statistic [45]. *P<0.01. Continuous line: observed mismatch distribution; dotted line: mismatch distribution expected under a pure demographic expansion model.

In total, 72 samples of *D. montalentii* were collected from 6 populations located on the main mountaintop of the island of Corsica, spanning the whole species range (Figure 1 and Table 1). Genomic DNA was extracted from tissue samples using the standard CTAB (cetyltrimethylammonium bromide) protocol [32]. Thereafter, 2 mitochondrial DNA fragments (cytochrome b, herein referred to as *cytb*; 12s ribosomal rRNA gene, herein referred to as 12*s*) were amplified by polymerase chain reactions (PCR), then purified and sequenced by Macrogen Inc. (www.macrogen.com). The primers used to amplify the *cytb* fragment were 494dismod (AACATYTCRGCGCTATGAAA) and cytdis (ATCGATTTAGAAGYTTRTTTTC); whereas primers 12SZ-L (AAAGGTTTGGTCCTAGCCTT) 12SF-H (CTTGGCTCGTAGTTGCCTGGCG) were used to amplify the 12*s* fragment [33]. Amplifications were carried out in a 25 µL volume containing MgCl_2_ (2.5 mM), the reaction buffer (5×, Promega), the 4 dNTPs (0.2 mM each), the 2 primers (0.2 µM each), the enzyme Taq polymerase (1 U, Promega), and 2 µL of DNA template. PCR cycling conditions were: one step at 94°C for 4 min followed by 35 cycles at 94°C for 1 min, 52°C (*cytb*) or 50°C (12*s*) for 1 min, 72°C for 1 min, and a single final step at 72°C for 10 min. All sequences obtained were deposited in GenBank (accession numbers: KC342972–KC343003; see Table S1).

**Table 1:** Geographic location of the 6 populations sampled of *Discoglossus montalentii*, number of individuals analysed (n), and haplotypes found in each population (h).

	Locality	Latidude N	Longitude E	n	h (n)
**1**	Stazzona	42°22′	09°22′	22	E1 (4); W3 (2); E2 (2); E3 (4); E4 (2); W4 (6); E6 (2);
**2**	Evisa	42°15′	08°48′	8	W1 (4); W2 (4);
**3**	Vizzavona	42°07′	09°07′	10	W2 (8); E5 (2);
**4**	Rio Pietra Piana	41°40′	09°12′	8	S1 (4); S2 (2); S6 (2);
**5**	L'Ospedale	41°38′	09°11′	14	S1 (10); S4 (2); S5 (2);
**6**	Sotta-Lieve	41°36′	09°08′	10	S1 (8); S3 (2);

### Data analysis

We used the program chromas 2.31 (Technelysium Ltd) to check the electropherograms by eyes and we produced a sequence alignment with clustalx [34]. Nucleotide (π) and haplotype (h) diversity [35] were calculated using the software dnasp 5 [36]. Sequence variation was analysed using mega 5.1. Since no differences were detected by a partition-homogeneity test [37] implemented in the software paup ^*^ 4.0b10 [38], the 2 mtDNA fragments were combined for all subsequent analyses.

The phylogenetic relationships among haplotypes were investigated using the median-joining algorithm for phylogenetic network estimation [39] as implemented in network 4.6.1.0 (www.fluxus-engineering.com), using the default option for all parameters in the analysis.

A simulated annealing procedure was used to identify groups of populations geographically homogeneous and maximally differentiated from each other. This analysis was conducted using samova [40] and a number of groups (*K*), ranging from 1 to 6. The optimal *K* was assessed as the one for which *F*_CT_ (i.e. the genetic variance due to between groups divergence) was the highest and statistically significant. To verify its consistency, we ran the analysis 5 times for each *K* value with 1000 independent annealing processes. The partition of the total genetic variation into its hierarchical components was evaluated through the analysis of molecular variance (AMOVA; [41]), which was carried out using arlequin 3.1 ([42], significance assessed by 1023 permutations). Three hierarchical levels were used: variance among groups, variance among populations within groups, and variance within population. The groups used in this analysis were those suggested by the samova analysis.

A mismatch distribution analysis [43] was carried out to investigate the historical demographic trend of the main mtDNA lineages as identified by the phylogenetic analysis, using the software arlequin. For each mtDNA lineage we also computed the *F*_S_ [44] and *R*_2_ [45] neutrality statistics using the software dnasp 5 [36], which appeared to be the most powerful test statistics against population growth in a recent comparison [45].

The time to the most recent common ancestor (TMRCA) of the main haplogroups was estimated using the distance-based least squares (LS) method recently described by Xia & Yang [46] as implemented in the software dambe [47]. The hypothesis of clock-like evolution of our sequences was tested by means of a likelihood ratio test as implemented in dambe. This test did not reject the molecular clock hypothesis. The best-fit model of nucleotide substitution was inferred using the Bayesian information criterion in modelgenerator 0.1.1 [48]. This software indicated that the HKY+Γ [49] model with a gamma distribution shape parameter of 0.11 was the best-fit one. We used a sequence drawn from *D.sardus* as outgroup (GenBank accessions: XXXX). A tree topology was obtained using the Neighbor-Joining algorithm (NJ) as implemented in mega, using the Tamura-Nei distance (which is the best approximation of the HKY distance available in mega) and 1000 bootstrap re-samplings to evaluate nodal support. The LS procedure in DAMBE was run using the NJ tree topology, the ‘softbound’ option and the ‘MLCompositeTN93’ genetic distance, as suggested by Xia & Yang [46]. The root age was set to 10 million years, as estimated by Zangari *et al.* [30] for the divergence time between *D. montalentii* and all other species of the genus *Discoglossus*. Finally, a bootstrap re-sampling procedure (with 1000 pseudoreplicates) was used to assess the standard deviation of the time estimates.

## Results

For all 72 individuals analysed, we obtained a fragment of 935 bp of the *cytb* gene and a fragment of 448 bp of the 12*s* gene. The combined dataset (overall 1383 bp in length) included 39 variable positions (29 parsimony informative) defining 16 haplotypes, whose geographic distribution among populations is shown in Table 1 (p-distances among haplotypes are shown in Table S1). Within the *cytb* gene fragment, no indels, stop codons, or non-sense codons were found. For the whole mtDNA dataset, haplotype diversity was 0.867 (±0.028 s.d.), while nucleotide diversity was 0.0073 (±0.0037 s.d.).

The phylogenetic network among haplotypes is shown in Figure 1B. Three main groups of haplotypes were observed (named clade S, W and E), and their geographic distributions are shown in Figure 1A. Clade S was geographically restricted to the southern portion of the species' range (populations 4–6), and it showed a star-like topology. Both clade W and clade E were observed in the central part of the island, but with a more western (populations 2, 3) and a more eastern (population 1) prevalence respectively. Neither clade W nor clade E showed a star-like shape within the haplotype network.

The samova analysis indicated 3 groups of populations (K = 3) as the best option of population grouping. Indeed, 68% of the total variation was explained at the among groups level with K = 3, while 51%, 64% and 57% was explained with K = 2, K = 4 and K = 5, respectively. One group comprised all of the populations carrying haplotypes from clade S (4, 5, 6), a second group included populations 2 and 3, carrying haplotypes of the clade W at higher frequencies, and the third group comprised just population 1, where clade E was the most represented. The amova analysis (Table 2) was performed by separating populations according to the best grouping indicated by samova. With this grouping option, 67.55% of the total genetic variation can be ascribed to the among-group level of variation (*F*_CT_ = 0.68), 0.91% to the among-population within-group level (*F*_SC_ = 0.03), and 31.55% to the within-population level (*F*_ST_ = 0.68).

**Table 2:** Analysis of molecular variation (amova) among populations of *Discoglossus montalentii*, grouped according to the results of the samova analysis.

Source of variation	Variance components	Percentage of variation	Fixation indices
**Among groups**	4.421 Va	67.55	*F*_CT_ 0.68*
**Among populations within groups**	0.059 Vb	0.91	*F*_SC_ 0.03*
**Within populations**	2.065 Vc	31.55	*F*_ST_ 0.68*

* P<0.01.

The mismatch distribution and the values of the demographic test statistics computed separately for the 3 clades, are shown in Figure 1C. Only the mismatch distribution of clade S showed the smooth wave predicted for a population that has recently undergone a sudden demographic expansion, while the distributions of clades W and E showed a ragged shape. A recent demographic growth for clade S was also suggested by the low and statistically significant value of the *R*_2_ statistic, and by the large negative and significant value of the *F*_S_ statistic.

**Figure 2:**
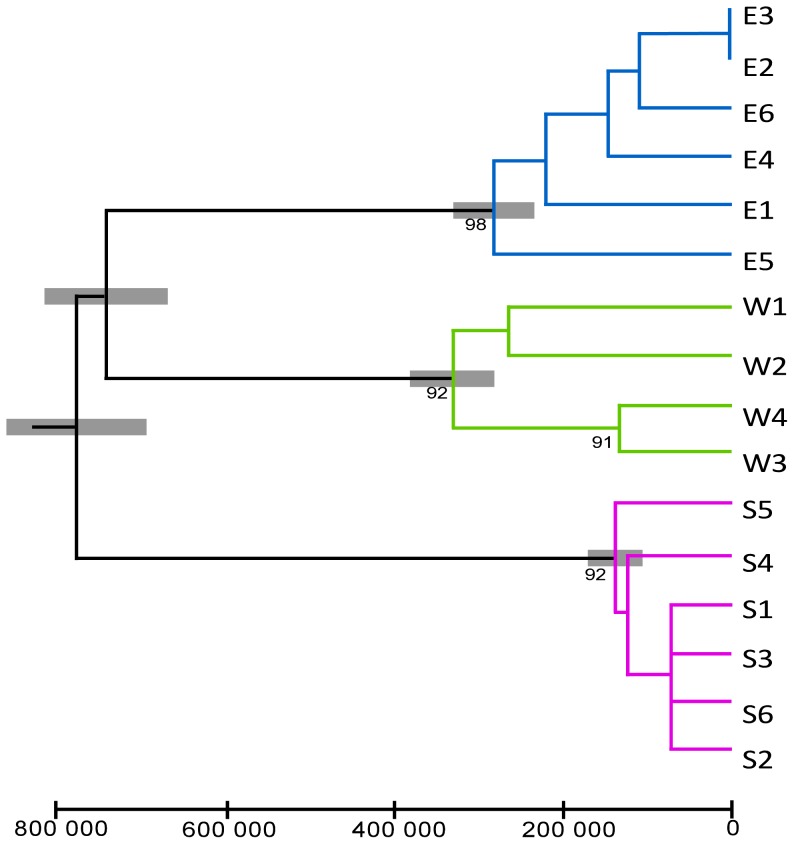
Chronogram of the main mtDNA lineages found in *Discoglossus montalentii*. Chronogram based on the NJ phenogram of the mtDNA haplotypes found in *D. montalentii*. Scale axis is in years. Bootstrap supports over 1000 pseudo-replicates of the NJ analysis are shown below the nodes (for supports >70%). Grey shading shows the standard deviations of the time estimates.

The chronogram showing the TMRCA of the main groups of haplotypes is shown in Figure 2. The split between clade S and the other clades was estimated to have occurred 777 000 (±166 000 s.d.) years before present (ybp), while the split time between clades E and W was estimated at 740 000 ybp (±146 000 s.d.). Finally, the TMRCAs for clades S, E and W were estimated at 138 000 ybp (±65 000 s.d.), 284 000 ybp (±96 000 s.d.), and 330 000 ybp (±100 000 s.d.) respectively.

## Discussion

What determines genetic diversity within species and populations is a longstanding and still largely open question in ecology and evolutionary biology (e.g. [50]–[54]). Island populations have been extensively used as model systems in this context, owing to the several simplifying assumptions that they allow. Being discrete, isolated and likely homogeneous entities, their genetic diversity has long been considered to be a function of island size (a correlate of population size), tempo and mode of island occupancy, and distance with neighbouring landmasses. Nevertheless, recent findings from intra-island phylogeographic studies are revealing more complex scenarios, suggesting that a prominent role in shaping current patterns of genetic diversity on islands are often determinated by microevolutionary processes acting within single islands [2], [6], [8]–[11]. The case of the Corsican endemic frog *D.montalentii*, as presented here, supports this revised view and exemplifies some of its major implications well (e.g. [12]–[17]).

The Corsican painted frog is a species with a substantially restricted distribution. Indeed, it is endemic to the Corsican island, where its distribution spans less than 5000 km^2^ [31], as it lives mainly in mountainous regions [55]. Moreover, its populations are spread with apparent continuity throughout its range. As a result we might expect a pattern of genetic homogeneity over the entire range of the species, without substantial phylogeographic discontinuities or divergent phylogroups. This expectation also forms the basis of the IUCN categorization of the conservation status of this species as ‘Near Threatened’, since “…it is not severely fragmented and it occurs at more than 10 locations” ([31]; although demographic data are still not available for the species).

Nevertheless, our data reveals a different scenario. Results of the network analysis showed a phylogeographic structure with 3 main groups, which is also supported by SAMOVA analysis (68% of the overall genetic variance is explained by variation among-groups). Each group was found to be geographically restricted to a portion of the species range: clade W in the north-western part, clade E in the north-eastern part, and clade S in the southern part of the island. Furthermore, the TMCRA analysis traced the split-time among these groups back to approximately the transition from Early to Middle Pleistocene.

The Early to Middle Pleistocene transition (globally dated at *c*.1.2-0.5 Ma [56]; often called the ‘mid-Pleistocene revolution’) was marked by crucial climate changes, particularly an increasing severity and duration of cold stages. This phase was characterized by an increase in global ice volume accompanied by cooler and drier atmospheric conditions. Moreover, throughout Europe, the first of the main glacial episodes occurred around 870000–880000 ybp, when severe paleoclimatic conditions were detected ([56] and references therein). This event has had profound effects on physical and biological landscapes [56]. As has often been inferred for montane species (see [57] and references therein), it is plausible that during this period *D. montalentii* was forced to move from mountain regions to lower valleys, where more suitable humidity and temperature conditions persisted [58], thus remaining fragmented, at least until the next interglacial period.

Historical demographic analyses indicated a recent demographic expansion for the southern clade S, but not for clades E and W. This parallels what has been recently found for *E. montanus*, another stream-breeding amphibian endemic to the Corsica mountains [12]. Paleoclimatic reconstructions for this area during the last glaciation [59] suggest significantly harsher climatic conditions in the northern rather than in the southern region of Corsica. Together with the topographic features of the island, this suggests that while northern populations may have been forced into mountain valleys by the altitudinal displacement occurring during the last glaciations, southern populations could have encountered wider areas of suitable climatic conditions, leading to the observed demographic expansion. Nevertheless, the co-presence of haplotypes belonging to clades E and W within samples 1 and 3 suggests that spatial expansions actually occurred at some point in time for these clades as well. As the genetic imprints of such expansions were not found, we can hypothesize that either they were not so pronounced as to leave detectable imprints (e.g. star-like phylogenies, bell-shaped mismatch distributions, significant test statistics as Fu's *F*s and Rasmos-Onsins and Rozas' *R*_2_), or alternatively, they may have occurred earlier in the history of these populations and their signatures were subsequentely erased.

The Pleistocene evolutionary history of *D. montalentii*, as we have inferred here, shows several similarities with what has been previously observed in other Corsican endemic species, particularly with respect to the spatial distribution of phylogeographic breaks, as well as a more structured pattern of variation in northern than in southern Corsica [12], [26], [27]. Nevertheless, geographic barriers that could account for limited dispersal (either current or historical) in northern Corsica were not apparent in these species, as is the case here. Together, this increases the plausibility that past climatic factors (i.e. range-wide rather than locally relevant processes) are most likely to have played a crucial role in shaping the distribution of populations and their genetic variability in this part of the island (see also [12], [26]).

Our results have significant implications for the species conservation. As mentioned above, it is currently considered as ‘Near Threatened’ according to the IUCN Red List. It has not been assigned to higher threat categories in part due to the belief that its populations are not significantly structured, a conclusion that is based on its ‘extent of occurrence’ (sensu IUCN Red List), since no data on population structure were available until now. The pattern emerging from our study does not fit with this prediction. Indeed, populations appeared to be fragmented into 3 main groups of ancient origin (Early-Middle Pleistocene). Moreover, most of the genetic diversity found was allocated among groups (68%), rather than within them. Although these results deserve to be explored further based on a panel of nuclear loci (e.g. to assess the impact of secondary contacts on the levels of population genetic diversity), the 3 groups identified here define 3 distinct evolutionary significant units (ESUs) that would warrant management for conservation [60]. Taking into account the observed fragmentation, we suggest reconsidering the IUCN Red List categorization of *D. montalentii*, as the whole set of data now available for this species could justify its elevation from ‘Near Threatened’ to ‘Vulnerable’ (see also [31], [61]).

### Concluding remarks

The data presented here add to the recently growing literature on genetic diversity of island populations in showing that microevolutionary processes acting within islands can be extremely relevant in explaining the current patterns of genetic diversity of island populations. Far from discounting the role of other processes (see e.g. [62]), these results suggest the need to integrate current theories and practices of island biogeography with the long neglected microevolutionary processes occurring within islands, and their imprints in the patterns of population genetic structure and diversity. This would be especially important from a conservation perspective: if island populations are no longer reliably approximated by homogeneous entities, island-wide panmixia is no longer a plausible expectation, island size is not a reliable proxy of effective population size, and the conservation status of island endemics should be carefully re-evaluated (on a case-by–case basis), as both demographic and genetic stochasticity are known to yield much more profound effects on structured, than on panmictic, populations (e.g. [63]).

## Supporting Information

Table S1 **Uncorrected pairwise divergence among the 16 composite haplotypes found in** ***D. montalentii*** **, and their genbank accession numbers.** Uncorrected pairwise divergence (p-distance) among the 16 composite haplotypes found in *D. montalentii*. Divergences for the 12S (448 bp) and CytB (935 bp) fragments are given below and above the diagonal respectively. The Genbank accession number of each haplotype is given below the distance matrix for both the 12S and the CytB fragments.
(DOCX)
