# Supplementary material for: Has Living on Islands Been So Simple? Insights from the Insular Endemic Frog Discoglossus montalentii
Source: PLoS One. 2013 Feb 5;8(2):e55735. doi: 10.1371/journal.pone.0055735 (PMC3564813; doi:10.1371/journal.pone.0055735)
Supplement: Table S1 — Uncorrected pairwise divergence among the 16 composite haplotypes found in D. montalentii , and their genbank accession numbers. Uncorrected pairwise divergence (p-distance) among the 16 composite haplotypes found in D. montalentii. Divergences for the 12S (448 bp) and CytB (935 bp) fragments are given below and above the diagonal respectively. The Genbank accession number of each haplotype is given below the distance matrix for both the 12S and the CytB fragments. (DOCX) [file pone.0055735.s001.docx]

**Table S1 Uncorrected pairwise divergence among the 16 composite haplotypes found in *D. montalentii*, and their genbank accession numbers.** Uncorrected pairwise divergence (p-distance) among the 16 composite haplotypes found in *D. montalentii*. Divergences for the 12S (448bp) and CytB (935bp) fragments are given below and above the diagonal respectively. The Genbank accession number of each haplotype is given below the distance matrix for both the 12S and the CytB fragments.

|  | S1 | S3 | S2 | S5 | S4 | W1 | W2 | S6 | E1 | W3 | E4 | E3 | E2 | E5 | W4 | E6 |
| --- | --- | --- | --- | --- | --- | --- | --- | --- | --- | --- | --- | --- | --- | --- | --- | --- |
| S1 | - | 0.001 | 0.000 | 0.001 | 0.001 | 0.011 | 0.010 | 0.001 | 0.012 | 0.009 | 0.012 | 0.011 | 0.013 | 0.012 | 0.007 | 0.012 |
| S3 | 0.000 | - | 0.001 | 0.002 | 0.002 | 0.012 | 0.011 | 0.002 | 0.013 | 0.010 | 0.013 | 0.012 | 0.014 | 0.013 | 0.009 | 0.013 |
| S2 | 0.000 | 0.000 | - | 0.001 | 0.001 | 0.011 | 0.010 | 0.001 | 0.012 | 0.009 | 0.012 | 0.011 | 0.013 | 0.012 | 0.007 | 0.012 |
| S5 | 0.000 | 0.000 | 0.000 | - | 0.002 | 0.012 | 0.011 | 0.002 | 0.013 | 0.010 | 0.013 | 0.012 | 0.014 | 0.013 | 0.009 | 0.013 |
| S4 | 0.002 | 0.002 | 0.002 | 0.002 | - | 0.012 | 0.011 | 0.002 | 0.013 | 0.010 | 0.013 | 0.012 | 0.014 | 0.013 | 0.009 | 0.013 |
| W1 | 0.007 | 0.007 | 0.007 | 0.007 | 0.009 | - | 0.005 | 0.012 | 0.014 | 0.006 | 0.014 | 0.013 | 0.015 | 0.014 | 0.005 | 0.014 |
| W2 | 0.007 | 0.007 | 0.007 | 0.007 | 0.009 | 0.000 | - | 0.011 | 0.013 | 0.005 | 0.013 | 0.012 | 0.014 | 0.013 | 0.004 | 0.013 |
| S6 | 0.000 | 0.000 | 0.000 | 0.000 | 0.002 | 0.007 | 0.007 | - | 0.013 | 0.010 | 0.013 | 0.012 | 0.014 | 0.013 | 0.009 | 0.013 |
| E1 | 0.007 | 0.007 | 0.007 | 0.007 | 0.009 | 0.009 | 0.009 | 0.007 | - | 0.012 | 0.004 | 0.003 | 0.001 | 0.000 | 0.011 | 0.004 |
| W3 | 0.009 | 0.009 | 0.009 | 0.009 | 0.011 | 0.002 | 0.002 | 0.009 | 0.007 | - | 0.012 | 0.011 | 0.013 | 0.012 | 0.001 | 0.012 |
| E4 | 0.009 | 0.009 | 0.009 | 0.009 | 0.011 | 0.011 | 0.011 | 0.009 | 0.002 | 0.009 | - | 0.001 | 0.005 | 0.004 | 0.011 | 0.002 |
| E3 | 0.009 | 0.009 | 0.009 | 0.009 | 0.011 | 0.011 | 0.011 | 0.009 | 0.002 | 0.009 | 0.000 | - | 0.004 | 0.003 | 0.010 | 0.001 |
| E2 | 0.009 | 0.009 | 0.009 | 0.009 | 0.011 | 0.011 | 0.011 | 0.009 | 0.002 | 0.009 | 0.000 | 0.000 | - | 0.001 | 0.012 | 0.005 |
| E5 | 0.004 | 0.004 | 0.004 | 0.004 | 0.007 | 0.007 | 0.007 | 0.004 | 0.002 | 0.009 | 0.004 | 0.004 | 0.004 | - | 0.011 | 0.004 |
| W4 | 0.013 | 0.013 | 0.013 | 0.013 | 0.016 | 0.007 | 0.007 | 0.013 | 0.011 | 0.004 | 0.013 | 0.013 | 0.013 | 0.013 | - | 0.011 |
| E6 | 0.011 | 0.011 | 0.011 | 0.011 | 0.013 | 0.013 | 0.013 | 0.011 | 0.004 | 0.011 | 0.002 | 0.002 | 0.002 | 0.007 | 0.013 | - |
| 12S | KC342988 | KC342989 | KC342990 | KC342991 | KC342992 | KC342993 | KC342994 | KC342995 | KC342996 | KC342997 | KC342998 | KC342999 | KC343000 | KC343001 | KC343002 | KC343003 |
| CytB | KC342972 | KC342973 | KC342974 | KC342975 | KC342976 | KC342977 | KC342978 | KC342979 | KC342980 | KC342981 | KC342982 | KC342983 | KC342984 | KC342985 | KC342986 | KC342987 |
